# *Stratocorticium sinensis* gen. et sp. nov. and *Cericium gloeocystidiatum* sp. nov. (*Cyphellaceae*, *Agaricales*) from East Asia

**DOI:** 10.3390/jof10100722

**Published:** 2024-10-17

**Authors:** Yu-Peng Zhang, Yue Li, Karen K. Nakasone, Shuang-Hui He

**Affiliations:** 1 School of Ecology and Nature Conservation, Beijing Forestry University, Beijing 100083, China; zypwell@163.com (Y.-P.Z.);; 2 Center for Forest Mycology Research, Northern Research Station, U.S. Forest Service, Madison, WI 53726, USA; karen.nakasone@usda.gov

**Keywords:** molecular phylogenetics, new species, new genus, species diversity, taxonomy, wood-decay fungi

## Abstract

*Cyphellaceae*, a small and under-studied family of *Agaricales*, includes mostly saprophytic taxa with varied basidiomes. In this study, we focus on wood-decay species with corticioid or stereoid basidiomes. Phylogenetic analyses of concatenated *ITS*-*nrLSU* sequences uncovered seven generic lineages of corticioid or stereoid fungi—*Acanthocorticium*, *Cericium*, *Chondrostereum*, *Cunninghammyces*, *Gloeostereum*, *Granulobasidium*, and *Stratocorticium* gen. nov. The genus *Cericium* is shown to be in the *Cyphellaceae* family, and two new species, *Cericium gloeocystidiatum* and *Stratocorticium sinensis*, are described from East Asia. Morphologically, *Ce*. *gloeocystidiatum* is characterized by resupinate basidiomes with smooth hymenophores, a dimitic hyphal system with clamped generative hyphae and micro-binding hyphae, cystidia with resinous-like or golden yellow contents, and ellipsoid basidiospores. *Stratocorticium* is monotypic, differing from *Cericium* by a trimitic hyphal system of clamped generative, micro-binding, and brown, thick-walled skeletal-like hyphae, clavate to cylindrical cystidia with homogenous, colorless contents, and hyphidia. Descriptions and illustrations are provided for the new taxa and *Cericium luteoincrustatum*, and a key to corticioid or stereoid genera in *Cyphellaceae* is included.

## 1. Introduction

*Cyphellaceae* Lotsy, typified by *Cyphella* Fr., is a small monophyletic family in the *Agaricales* containing taxa with agaricoid, cyphelloid, clavarioid/typhuloid, pleurotoid, corticioid or stereoid basidiomes [1]. Five wood-decay genera with corticioid or stereoid basidiomes in the family include *Acanthocorticium* Baltazar, Gorjón & Rajchenb, *Chondrostereum* Pouzar, *Cunninghammyces* Stalpers, *Gloeostereum* S. Ito & S. Imai, and *Granulobasidium* Jülich [2,3]. He et al. [4] also included three corticioid genera in the *Cyphellaceae* family, namely *Gloeocorticium* Hjortstam & Ryvarden, *Hyphoradulum* Pouzar, and *Thujacorticium* Ginns. *Gloeocorticium* has not been sequenced, but it is similar to *Radulomyces* M.P. Christ. (*Radulomycetaceae*, *Agaricales*) by sharing clavate basidia and ellipsoid basidiospores with a distinct apiculus [5]. *Hyphoradulum* is in the *Cystostereaceae* (*Agaricales*) family and placed in synonymy under *Crustomyces* Jülich by molecular evidence [6]. The placement of the monotypic *Thujacorticium* is unknown because sequence data are lacking, but it shares some morphological features found in *Cerocorticium* Henn. (*Pterulaceae*, *Agaricales*) and *Hyphoderma* Wallr. (*Hyphodermataceae*, *Polyporales*), such as the monomitic hyphal system with clamped generative hyphae, the relatively large clavate to subcylindrical basidia, and large ellipsoid to cylindrical basidiospores [2,7].

The five corticoid or stereoid genera in the *Cyphellaceae* are monotypic or contain up to three species but are morphologically well characterized and phylogenetically distinct [1]. *Acanthocorticium* is monotypic and characterized by resupinate, cartilaginous basidiomes, dextrinoid acanthophyses, apically echinulate halocystidia with a resinous cap, and cyanophilous, globose basidiospores [3]. *Chondrostereum* is a well-known genus with effused to effused-reflexed basidiomes and often with a pigmented hymenophore, leptocystidia, vesicles, and cylindrical basidiospores [8,9]. *Cunninghammyces* includes two species and is characterized by soft, pellicular to membranous basidiomes, terminal or lateral basidia, and globose, thick-walled, echinulate, and inamyloid basidiospores [10]. *Gloeostereum*, typified by *Gl. incarnatum* S. Ito & S. Imai and distributed in northeast Asia, is a well-known edible fungus. Its pileate basidiomes are gelatinous and contain gloeohyphae and gloeocystidia [11]. The monotypic *Granulobasidium* is characterized by resupinate basidiomes, long tubular basidia with granular contents, thick-walled, rugose, cyanophilous basidiospores, and abundant chlamydospores [9].

Previous studies showed that some clades of *Agaricales* contained abundant wood-decay fungi, especially corticioid species [2,12,13]. Li et al. [12] intensively studied the taxonomy and phylogeny of *Cystostereaceae* and recovered a new genus and five new species. However, the species diversity, taxonomy, and phylogeny of the corticioid fungi in other clades of *Agaricales* are still under-investigated and urgent for comprehensive studies. A lot of wood-decay specimens recently collected from China were shown to belong to *Cyphellaceae* based on sequence blast results, but some of them could not be accurately identified. Thus, in the present study, we performed intensive phylogenetic analyses by focusing on the sequence data of corticioid and stereoid specimens. The results of this study will help us to understand the phylogenetic relationship and a reasonable taxonomic system of *Cyphellaceae* or even the *Agaricales.*

## 2. Materials and Methods

### 2.1. Specimen Collection

Specimens were collected in various nature reserves and forest parks of different types of forests in China by the authors during the rainy seasons, usually from June to October. In situ photographs of specimens were captured using a Canon EOS 70D camera (Canon Corporation, Tokyo, Japan). Fresh specimens were dried with a portable dryer (Evermat, Helsinki, Finland). Dried specimens were placed in a freezer at minus 40 °C for two weeks to kill the insects before proceeding with morphological and molecular studies. Voucher specimens were deposited at the herbaria of Beijing Forestry University, Beijing, China (BJFC), and the Center for Forest Mycology Research, Madison, WI, USA (CFMR).

### 2.2. Morphological Studies

Thin, freehand sections from dried basidiomes were mounted in 2% (weight/volume) aqueous potassium hydroxide (KOH) and 1% (*w*/*v*) aqueous phloxine. The amyloidity and dextrinoidity of hyphae and basidiospores were determined using Melzer’s reagent (IKI), while the cyanophily of hyphal and basidiospore walls were observed in 1% (*w*/*v*) cotton blue in 60% (*w*/*v*) lactic acid (CB). A Nikon Eclipse 80i (Nikon Corporation, Tokyo, Japan) or an Olympus BH2 microscope (Evident Corporation, Tokyo, Japan) was used for microscopic examinations at magnifications up to 1000×. Microscopic structures of holotypes were shown in photos taken using a camera or drawn using a drawing tube. The following abbreviations were used: IKI– = neither amyloid nor dextrinoid, CB– = acyanophilous, L = mean spore length, W = mean spore width, Q = L/W ratio, and n (a/b) = number of spores (a) measured from the number of specimens (b). The color codes and names follow Kornerup and Wanscher [14].

### 2.3. DNA Extraction and Sequencing

The extraction of total genomic DNA from the dried specimens was performed using a CTAB plant genomic DNA extraction kit, DN14 (Aidlab Biotechnologies Co., Ltd., Beijing, China), according to the manufacturer’s instructions. The *ITS* region and the D1-D2 region of the nucleic ribosomal *LSU* (*nrLSU*) were amplified by the Polymerase Chain Reaction (PCR) using the primer pairs *ITS5*/*ITS4* and *LR0R*/*LR7* [15,16]. The PCR procedures for the *ITS* region involved an initial denaturation at 95 °C for 3 min, followed by 34 cycles of denaturation at 94 °C for 40 s, annealing at 54 °C for 45 s, and extension at 72 °C for 1 min, with a final extension at 72 °C for 10 min. The PCR procedures for the *nrLSU* region involved an initial denaturation at 94 °C for 1 min followed by 34 cycles of denaturation at 94 °C for 30 s, annealing at 50 °C for 1 min, and extension at 72 °C for 1.5 min, with a final extension at 72 °C for 10 min. The PCR products were purified and sequenced at the Beijing Genomics Institute (BGI), Beijing, China, with the same primer sanger method using the ABI 3730XL sequencer (Applied Biosystems, Foster City, CA, USA). Newly generated sequences were deposited in GenBank (https://www.ncbi.nlm.nih.gov/, accessed on 31 January 2021). BioEdit v.7.0.5.3 [17] and Geneious Basic v.11.1.15 [18] were used to review the chromatograms and for contig assembly.

### 2.4. Phylogenetic Analyses

Previous studies [1,3] were consulted to determine taxa to include in the concatenated *ITS*-*nrLSU* sequence dataset of the *Cyphellaceae* (Table 1). *Flammulina velutipes* (Curtis) P. Karst was selected as the outgroup [1]. Sequences of *ITS* and *nrLSU* were aligned separately using MAFFT v.74 (http://mafft.cbrc.jp/alignment/server/, accessed on 1 May 2024) [19] with the G-INS-I iterative refinement algorithm and optimized manually in BioEdit v.7.0.5.3. The separate alignments were then concatenated using Mesquite v.3.5.1 [20]. The final alignments and the topologies were deposited in TreeBase (http://treebase.org/treebase-web/home.html, submission ID: 31374, accessed on 7 May 2024). Maximum parsimony (MP), maximum likelihood (ML) analyses, and Bayesian inference (BI) were carried out by using PAUP* v.4.0b10 [21], RAxML v.8.2.10 [22] and MrBayes 3.2.6 [23], respectively. The best-fit substitution model was estimated with PhyloSuite v1.2.3. [24]. Four Markov chains were run for 3,000,000 generations for the dataset until the split deviation frequency value was lower than 0.01.

## 3. Results

### 3.1. Phylogenetic Studies

**Figure 1:**
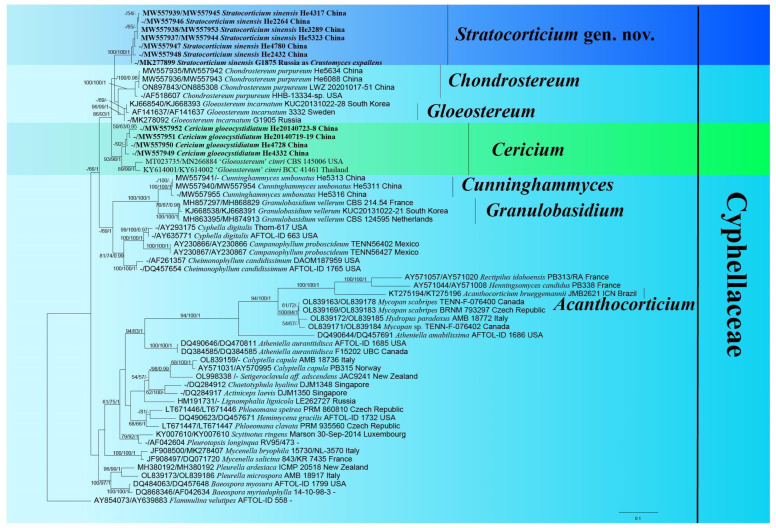
Phylogeny of *Cyphellaceae* generated by ML analyses based on combined *ITS*-*nrLSU* sequences. Branches are labeled with parsimony bootstrap values (≥50%, first), likelihood bootstrap values (≥50%, second), and Bayesian posterior probabilities (≥0.95, third). New taxa are highlighted and set in bold.

The concatenated *ITS*-*nrLSU* dataset contained 42 *ITS* and 56 *nrLSU* sequences from 60 samples representing 34 ingroup taxa and the outgroup taxon (Table 1) with an aligned length of 1516 characters, of which 821 were parsimony-informative. PhyloSuite suggested GTR + F + I + G4 as the best-fit model of nucleotide evolution for *ITS* and *nrLSU* regions, respectively. The average standard deviation of split frequencies of BI was 0.006497 at the end of the run. The MP and BI analyses resulted in almost identical tree topologies with the ML analysis. The ML tree is shown in Figure 1.

In the tree (Figure 1), seven generic lineages of corticioid and stereoid fungi were recovered—*Acanthocorticium*, *Cericium*, *Chondrostereum*, *Cunninghammyces*, *Gloeostereum*, *Granulobasidium*, and *Stratocorticium* gen. nov. *Acanthocorticium* nested in a lineage with cyphelloid and agaricoid basidiomes—*Rectipilus idahoensis* (W.B. Cooke) Agerer, *Mycopan*, *Hydropus paradoxus* M.M. Moser, and *Henningsomyces candidus* (Pers.) Kuntze (94/100/1). *Cunninghammyces* and *Granulobasidium* clustered together (100/100/1) in a clade sister to the *Campanophyllum*, *Cheimonopohyllum*, and *Cyphella* clades. *Stratocorticium*, *Chondrostereum*, *Gloeostereum*, and *Cericium* clustered together in a large clade (86/93/1). Within the *Cericium* lineage, *Ce. gloeocystidiatum* was sister to ‘*Gloeostereum*’ *cimri* S.A. Ahmed, D.A. Stevens & de Hoog (93/98/1). The sequence of *Crustomyces expallens* G1875 (MK277899) from the Far East of Russia nested within the *Stratocorticium* lineage (100/100/1).

### 3.2. Taxonomy

***Cericium*** Hjortstam, Mycotaxon 54: 184, 1995, emended.

Type species: *Amethicium luteoincrustatum* Hjortstam & Ryvarden, Mycotaxon 25(2): 542, 1986.

Basidiomes annual, resupinate, effused, adnate, thin to 1 mm thick, ceraceous, coriaceous, or crustaceous. Hymenophores were smooth to tuberculate, sometimes cracked, with a margin thinning out or abrupt, adnate, distinct. The hyphal system dimitic, generative hyphae clamped, micro-binding hyphae abundant, aseptate, thick-walled, and frequently branched. Cystidia of two or three types: originating from hymenium, short, subfusiform with acute apices, from subhymenium and hymenium, cylindric to fusiform, dark yellow, with resinous-like contents, or capitate with stalk. Basidia clavate, colorless, thin-walled, smooth, with four sterigmata and a basal clamp connection. Basidiospores ellipsoid or cylindrical, colorless, thin-walled, smooth, IKI–, CB–.

Notes—*Cericium* is characterized by ceraceous basidiomes with a smooth to tuberculate hymenophore, a dimitic hyphal system, and two or three types of cystidia. It is segregated from *Amethicium* Hjortstam, typified by *Am*. *rimosum* Hjortstam, which develops violet basidiomes and lacks cystidia [40]. Sequences of the type species of both genera were not available, so their classification is uncertain [2]. An undescribed species from southern China nested within the *Cyphellaceae* and is morphologically similar to *Ce*. *luteoincrustatum* with minor differences; the two species are described and illustrated below.

**Figure 2:**
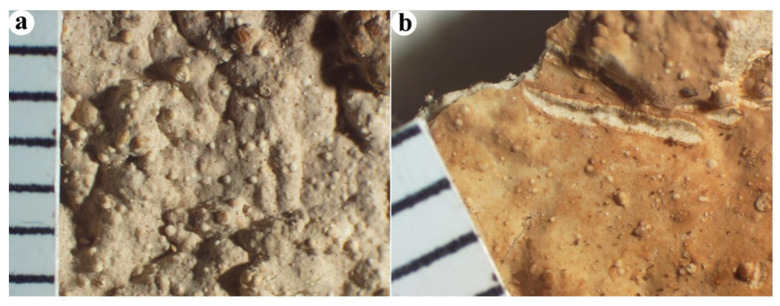
Basidiomes of *Cericium luteoincrustatum* (from the holotype, F-450396). (**a**,**b**) Hymenophore.

**Figure 3:**
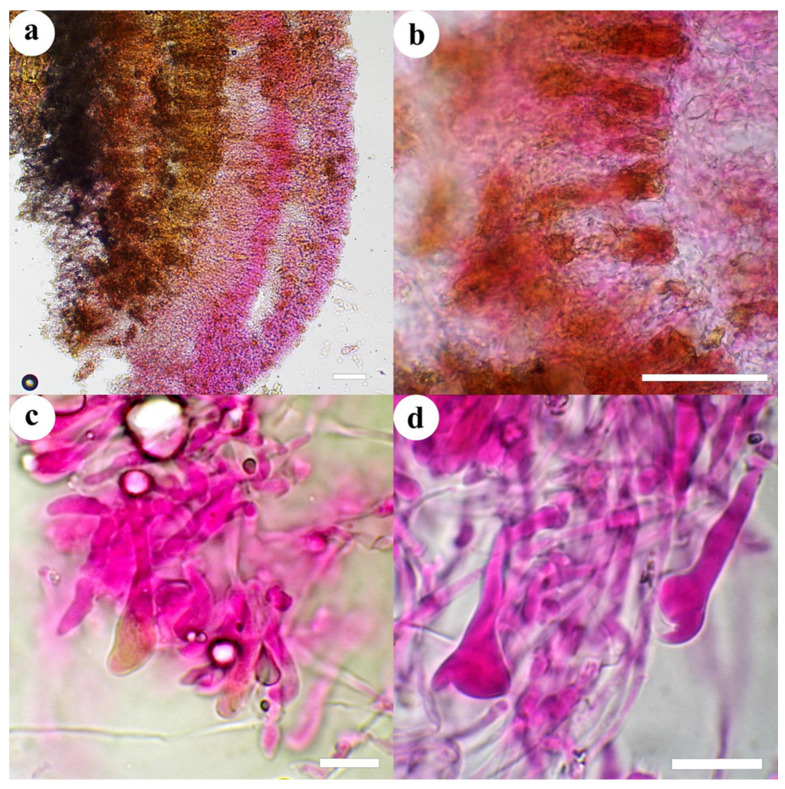
*Cericium luteoincrustatum* (from the holotype F-450396). Scale bars: (**a**) = 50 µm; (**b**–**d**) = 20 µm. (**a**) Part of a vertical section through basidiome; (**b**) Close-up of a vertical section through basidiome; (**c**) hymenium with subfusiform hymenial cystidia and clavate cystidia with yellow, resinous-like contents; (**d**) partially collapsed, capitate cystidia with stalk from subhymenium.

***Cericium luteoincrustatum*** (Hjortstam & Ryvarden) Hjortstam, Mycotaxon 54: 185, 1995. Figure 2 and Figure 3.

Fruiting body—Basidiome resupinate, widely effused, adnate, up to 500 µm thick, soft, crustaceous. Hymenophore smooth to irregularly studded with numerous, small, globular tubercules, ceraceous, grayish orange [5B (3–5)] with a grayish cast, sparsely cracked; context white to pale yellow, soft, fibrous, cottony; margin distinct, abrupt, adnate, thinning out, cream-colored, fibrillose.

Microscopic structures—Hyphal system dimitic with clamped generative hyphae and micro-binding hyphae. Subiculum at first a loose tissue up to 150 µm thick, composed primarily of loosely intertwined, encrusted subicular hyphae and micro-binding hyphae, eventually micro-binding hyphae becoming dominant; subicular hyphae 2–3 µm in diam., clamped, moderately branched, walls colorless, thin to thickened, often heavily encrusted with colorless or pale yellow crystals; micro-binding hyphae 0.5–2 µm wide, non-septate, frequently branched at right angles, attenuating, usually lacking a lumen, non-staining, walls colorless, thick, smooth. Subhymenium thickening, up to 350 µm thick, sometimes stratified, lower subhymenium, up to 300 µm thick, a dense, dark brownish yellow, agglutinated tissue of indistinct cystidial and hyphal remnants, upper or current subhymenium layer, up to 150 µm thick, a less dense, partially agglutinated tissue of hyphae and embedded cystidia empty or with dark yellow, oil-like contents; subhymenial hyphae 1.5–2.5 µm in diam., clamped, frequently branched, walls colorless, thin, smooth; micro-binding hyphae as described above. Hymenium a dense palisade of cystidia and basidia. Cystidia of three types: (1) originating in hymenium, numerous, subulate, nearly papillate or slightly tapering toward apex, 16–18 × 3.5–4 µm, clamped at the base, with homogeneous contents, walls colorless, thin, smooth; (2) originating in subhymenium, numerous, clavate, subfusiform, or cylindrical, apex obtuse or acute, 30–130 × 5–8 µm, clamped at the base, embedded, sometimes exserted, often filled with dark yellow, oil-like material, negative in sulfovanillin, walls colorless, thin to slightly thickened, smooth; (3) originating in subhymenium, embedded, scattered, capitate with a slender stalk, 24–40 × 8–12, stalk 2–3 µm diam., cap often partially collapsed, clamped at base, staining in phloxine, walls colorless, thin, smooth. Basidia clavate, 18–23 × 4–5 µm, clamped at base, 4-sterigmate, walls colorless, thin, smooth. Basidiospores cylindrical to broadly cylindrical, with walls colorless, thin, smooth, IKI–, CB–, 4–5.2 × 2.2–3 µm, L = 4.6 µm, W = 2.8 µm, Q = 1.7 (n = 30/1).

Specimen examined—Argentina, Missiones Province, Iguazu National Park, Cataratas de Iguazu, on deciduous wood, 1–5 March 1982, L. Ryvarden 19529, F-450396 (O, holotype).

Substrate—on wood and bark of angiosperms.

Distribution—Argentina.

Notes—*Cericium luteoincrustatum* is characterized by an effused, smooth to irregular basidiome studded with numerous, small tubercules, a dimitic hyphal system with microbinding hyphae, subulate hymenial cystidia, cylindrical to subfusiform cystidia with dark yellow contents, and capitate cystidia with a long stalk, small clavate basidia, and cylindric basidiospores. The basidiome has two distinct layers—a fertile upper layer that is thin and ceraceous and a wider, lower layer that is soft and white. The hymenial cystidia and capitate cystidia are described herein for the first time.

In many ways, it is morphologically similar to *Corticium expallens* Bres. that has a stratose basidiome of similar construction, micro-binding hyphae, and cylindrical cystidia [41]. The basidia and basidiospores of *Corticium expallens* are much larger than *Ce*. *luteoincrustatum*, and *Co*. *expallens* lacks cystidia with dark yellow contents.

**Figure 4:**
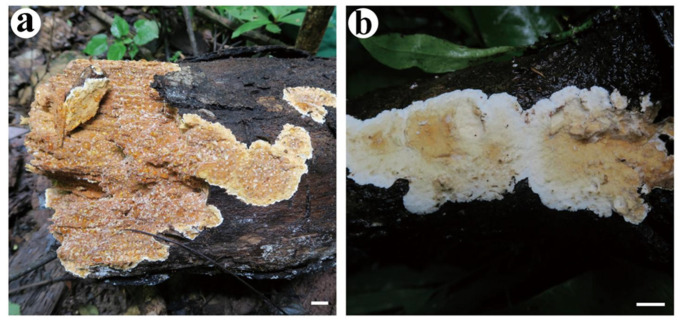
Basidiomes of *Cericium gloeocystidiatum*. Scale bars: (**a**,**b**) = 1 cm. (**a**) He 4332 (BJFC 023774, holotype); (**b**) He 4725 (BJFC 024244).

**Figure 5:**
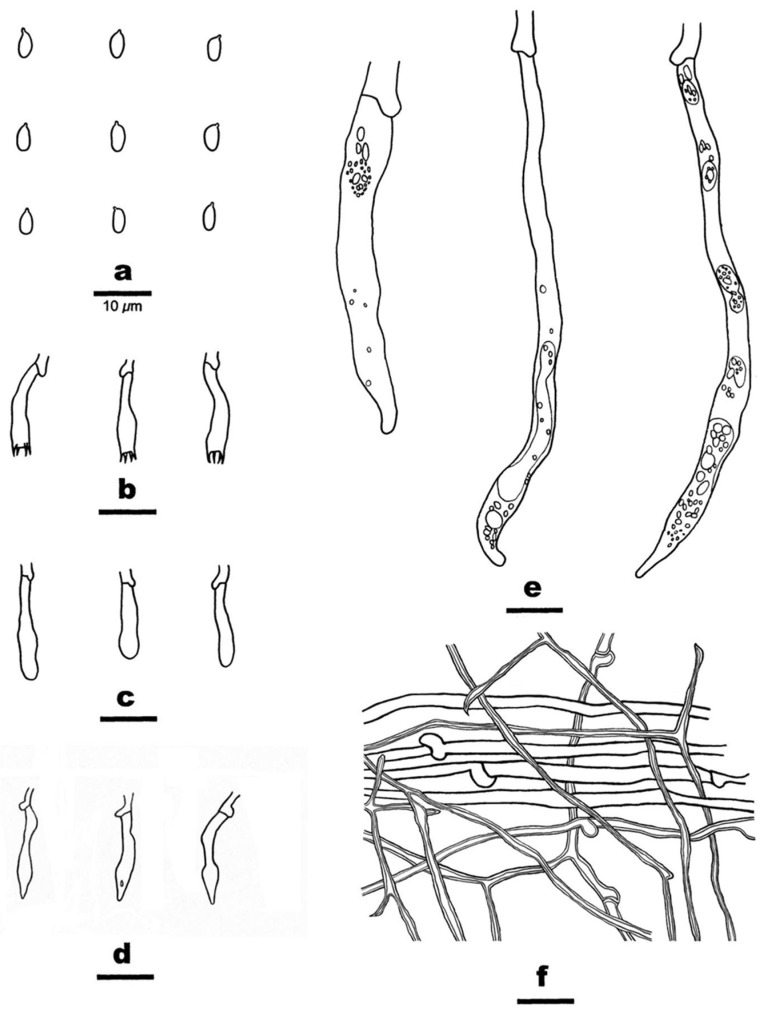
Microscopic structures of *Cericium gloeocystidiatum* (from the holotype He 4332). Scale bars: (**a**–**f**) =10 µm. (**a**) Basidiospores; (**b**) Basidia; (**c**) Basidioles; (**d**) subfusiform cystidia from hymenium; (**e**) Cystidia with dark yellow, resinous-like contents; (**f**) Generative and micro-binding hyphae from subiculum.

***Cericium gloeocystidiatum*** S.H. He, Y.P. Zhang & Nakasone, **sp. nov.** Figure 4 and Figure 5.

MycoBank: MB854276.

Type—China, Jiangxi Province, Lianping County, Jiulianshan Nature Reserve, on fallen angiosperm trunk, 13 August 2016, S.H. He, He 4332 (BJFC 023774, holotype; CFMR, isotype, dried specimen).

Etymology—*gloeocystidiatum* (Lat.) refers to the long and distinct gloeocystidia.

Fruiting body—Basidiomes annual, resupinate, effused, adnate, separable from substrate, gelatinous when fresh, becoming coriaceous or crustaceous after drying, first as small patches, later confluent up to 17 cm long, 4 cm wide, up to 400 µm thick in section. Hymenophore smooth or slightly tuberculate, grayish orange (6B6) to brownish orange (6C5), unchanged in KOH, cracked; context white to pale cream; margin thinning out or abrupt, adnate, distinct, white or paler than hymenophore.

Microscopic structures—Hyphal system dimitic; generative hyphae bearing clamp connections; micro-binding hyphae abundant in the subiculum. Subiculum up to 350 µm thick, a non-agglutinated tissue of densely interwoven hyphae; generative hyphae colorless, thin- to slightly thick-walled, smooth, frequently branched and septate, 2–3 µm in diam.; micro-binding hyphae abundant, dominant in subiculum, colorless, distinctly thick-walled to nearly solid, smooth, frequently branched, aseptate, 0.6–1 µm in diam. Subhymenium up to 25 µm thick, a dense, often partially agglutinated tissue. Cystidia abundant of two kinds: (1) cylindric then tapering to subacute apex, sinuous, colorless, thin-walled, smooth, embedded, often filled with dark yellow contents, if originating in subiculum 60–138 × 2–7 µm, those in hymenium 15–30 × 3.2–4.5 µm; (2) subhymenial in origin, subfusiform with acute apices, thin- to slightly thick-walled, sometimes with homogeneous, colorless contents, 12–20 × 2–3.8 µm. Basidia clavate, colorless, thin-walled, smooth, with four sterigmata and a basal clamp connection, 10–18 × 2.5–4.5 µm. Basidiospores cylindric, with an apiculus, colorless, thin-walled, smooth, IKI–, CB–, 4–5 (–5.5) × (1.8–) 2–2.3 µm, L = 4.4 µm, W = 2.1 µm, Q = 2–2.2 (n = 90/3).

Additional specimens examined—China, Guangxi Autonomous Region, Huanjiang County, Mulun National Nature Reserve, on rotten angiosperm trunk, 10 July 2017, S.H. He, He 4728 (BJFC 024247); Yunnan Province, Mengla County, Wangtianshu Scenic Spot, on fallen angiosperm branch, 19 July 2014, S.H. He, He 20140719-19 (BJFC 019146); Banna Botanical Garden, on fallen angiosperm branch, 23 July 2014, S.H. He, He 20140723-8 (BJFC 019187).

Substrate—on fallen trunks or branches of angiosperms.

Distribution—southern China.

Notes—*Cericium gloeocystidiatum* is characterized by resupinate basidiomes with a smooth hymenophore and somewhat gelatinous texture when fresh, a dimitic hyphal system with clamped generative and micro-binding hyphae, two kinds of cystidia, small basidia, and ellipsoid basidiospores. In Figure 1, four samples of *Ce. gloeocystidiatum* formed a strongly supported lineage sister to ‘*Gloeostereum*’ *cimri* that was isolated from the human pulmonary cyst and lacks a teleomorph [31]. *Cericium luteoincrustatum* and *Ce*. *gloeocystidiatum* share basidiome, hymenophore, and microscopic features, but the former differs in also developing capitate cystidia. Morphologically, *Gloeostereum incarnatum* S. Ito & S. Imai and *Ce. gloeocystidiatum* have gelatinous basidiomes, cystidia, and clamped generative hyphae in common, but the former differs in developing pileate basidiomes, a monomitic hyphae system, hyphidia, and slightly larger basidiospores (6–8.5 × 2.7–3.6 µm) [11].

***Stratocorticium*** S.H. He, Y.P. Zhang & Nakasone, **gen. nov.**

MycoBank: MB854277.

Type species—*Stratocorticium sinensis* S.H. He, Y.P. Zhang & Nakasone.

Etymology—“*strato*-”: stratified, refers to the stratified and thickening subhymenium; “*Corticium*”, a corticioid genus, refers to the similarity in basidiome morphology.

Basidiomes annual, resupinate, effused, closely adnate, inseparable from the substrate, coriaceous to soft corky. Hymenophore smooth to slightly tuberculate, light brown to brown, unchanged in KOH, sometimes sparsely cracked after drying; context stratified, dark gray to black with thin, white lines; margin abrupt or thinning out, white or slightly paler than hymenophore surface when juvenile, becoming indistinct with age. Hyphal system trimitic; generative hyphae clamped, brown, thick-walled, skeletal-like hyphae, and colorless, frequently branched micro-binding hyphae. Cystidia of two kinds: (1) clavate to cylindrical, colorless, thin-walled; (2) capitate, with short stalk, thin-walled and colorless at first, then walls slightly thickened, brown. Hyphidia simple or branched with several knob-like branches, walls thin, colorless at first then thick-walled, brown. Basidia clavate, with one to several constrictions, thin-walled, with four sterigmata and a basal clamp connection. Basidiospores ellipsoid, colorless, thin-walled, smooth, IKI–, CB–.

Notes—*Stratocorticium* is characterized by resupinate, coriaceous, thick basidiomes with smooth to tuberculate hymenophores, a thickening subhymenium, a trimitic hyphal system with clamped generative, brown, thick-walled skeletal-like, and microbinding hyphae, clavate to cylindrical cystidia with homogenous contents, capitate cystidia, hyphidia, and ellipsoid basidiospores. In the phylogenetic tree, *Stratocorticium* is closely related to *Chondrostereum* and *Gloeostereum*. *Chondrostereum* can be easily distinguished from *Stratocorticium* by its effused to effused-reflexed basidiomes, pinkish to violaceous hymenophore, monomitic hyphal system, and vesicles [8,9]. The development of gloeohyphae and gloeocystidia readily differentiates *Gloeostereum* from *Stratocorticium* [11]. *Cericium* and *Stratocorticium* have similar basidiome and hymenophore features with clamped generative hyphae, microbinding hyphae, and vesicles or capitate cystidia, but the former differs in developing cystidia with dark yellow, resinous-like contents and lacks skeletal-like hyphae and hyphidia.

**Figure 6:**
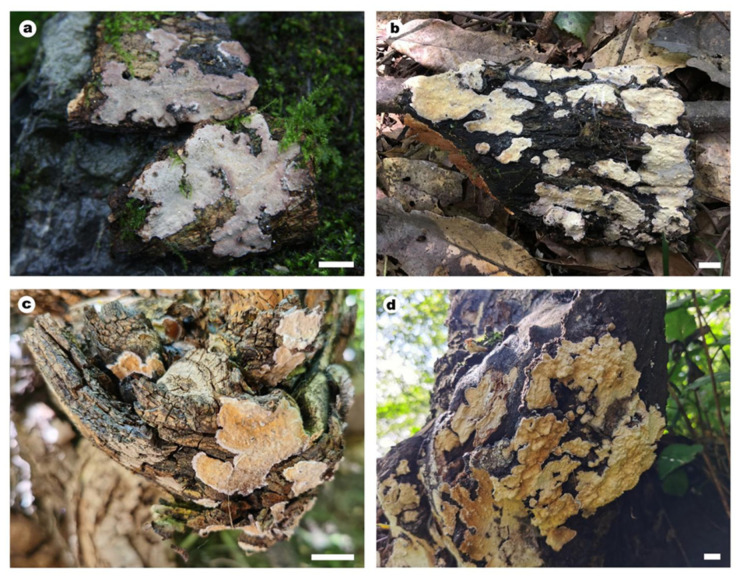
Basidiomata of *Stratocorticium sinensis*. Scale bars: (**a**–**d**) = 1 cm. (**a**) He 2264 (BJFC 020718); (**b**) He 5349 (BJFC 024867); (**c**) He 6875 (BJFC 033824); (**d**) He 6895 (BJFC 033844).

**Figure 7:**
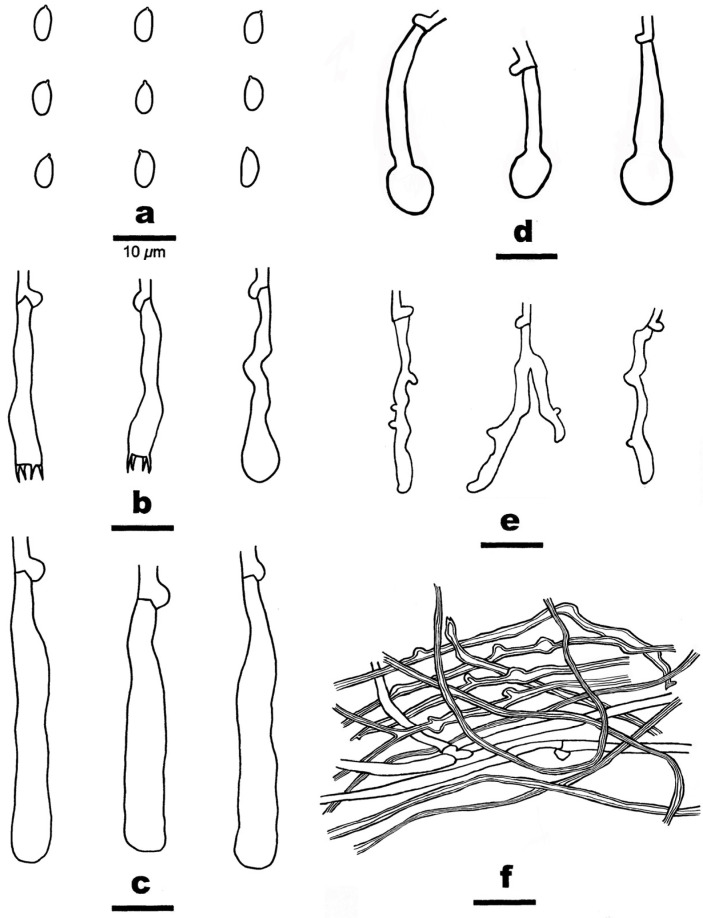
Microscopic structures of *Stratocorticium sinensis.* (from the holotype He 3289). Scale bars: (**a**–**f**) = 10 µm. (**a**) Basidiospores; (**b**) Basidia and a basidiole; (**c**) Embedded cystidia; (**d**) Capitate cystidia, (**e**) Hyphidia; (**f**) Generative, micro-binding, and skeletal-like hyphae from subhymenium.

***Stratocorticium sinensis*** S.H. He, Y.P. Zhang & Nakasone, **sp. nov.** Figure 6 and Figure 7.

MycoBank: MB855647.

Type—China, Yunnan Province, Lushui County, Gaoligongshan Nature Reserve, on the dead angiosperm tree, 28 November 2015, S.H. He, He 3289 (BJFC 021684, holotype, dried specimen).

Etymology—refers to the type locality in China.

Fruiting body—Basidiomes annual, resupinate, widely effused, closely adnate, inseparable from the substrate, corneous to soft corky, first as small patches, later confluent up to 20 cm long, 8 cm wide, up to 3 mm thick in section, and distinctly stratified. Hymenophore smooth to slightly tuberculate, light brown (7D4) or brown (7E4), unchanged in KOH, not cracked or sparsely and deeply cracked after drying; margin abrupt or thinning out, white to slightly paler than hymenophore surface when juvenile, becoming indistinct with age. Context stratified, dark gray to nearly black, interspersed with several thin, white stripes.

Microscopic structures—Hyphal system trimitic; generative hyphae bearing clamp connections, brown, skeletal-like hyphae, and micro-binding hyphae. Subiculum thin; generative hyphae colorless, thin- to slightly thick-walled, smooth, interwoven, more or less parallel to the substrate, frequently branched, occasionally septate, 2–3 µm in diam; micro-binding hyphae abundant, colorless, distinctly thick-walled to nearly solid, smooth, frequently branched, aseptate, 0.5–1.5 µm in diam. Subhymenium distinct, thickening, stratified with age, with layers of generative and micro-binding hyphae as in subiculum interspersed with zones of brown, skeletal-like hyphae with thick walls, occasionally branched at right angles, smooth or encrusted with colorless crystals, IKI–, CB–, 2–3 µm diam. Hymenium composed of hyphidia, cystidia, and basidia. Hyphidia cylindric, often with short, knobby branching at apex, colorless and thin-walled at first, then brown, up to 1 µm thick, smooth, IKI–, CB–,12.5–35 × 1.5–3.3 µm. Cystidia of two kinds: (1) embedded, clavate to cylindrical, colorless, thin-walled, 37–55 × 5–6.5 µm; (2) capitate with short stalk, thin-walled and colorless at first, then walls slightly thickened, brown, 10–20 × 5.5–7.5 µm. Basidia clavate, with one to several constrictions, thin-walled, smooth, with four sterigmata and a basal clamp connection, 21–36.5 × 4–5 µm. Basidiospores cylindric, with an apiculus, colorless, thin-walled, smooth, IKI–, CB–, 5–6 × 2–2.8 (–3) µm, L = 5.5 µm, W = 2.3 µm, Q = 2.3–2.4 (n = 90/3).

Additional specimens examined—China, Beijing, Huairou District, Labagou Forest Park, on *Acer* trunk, 28 October 2018, S.H. He, He 5706 (BJFC 030573); on *Quercus* stump, 25 July 2020, S.H. He, He 6875 (BJFC 033824); Mentougou District, Xiaolongmen Forest Park, on *Ailanthus* stump, 28 July 2020, S.H. He, He 6895 (BJFC 033844); Lingshan Nature Reserve, on dead angiosperm branch, 10 September 2022, S.H. He, He 7470 (BJFC 038606); Guizhou Province, Libo County, Maolan Nature Reserve, on angiosperm stump, 11 July 2017, S.H. He, He 4780 (BJFC 024297, CFMR); Hunan Province, Shimen County, Hupingshan Nature Reserve, on angiosperm stump, 5 June 2015, S.H. He, He 2264 (BJFC 020718); on the living *Acer oblongum*, 6 June 2015, S.H. He, He 2280 (BJFC 020735), and He 2282 (BJFC 020737); Jiangxi Province, Lianping County, Jiulianshan Nature Reserve, on dead *Cinnamomum camphora* branch, 13 August 2016, S.H. He, He 4317 (BJFC 023759, CFMR); Ningxia Autonomous Region, Jingyuan County, Liupanshan Forest Park, on base of living angiosperm tree, 4 August 2015, S.H. He, He 2432 (BJFC 020885); Yunnan Province, Binchuan County, Jizu Mountain Scenic Spot, on angiosperm stump, 26 November 2015 S.H. He, He 3197 (BJFC 021592); 28 October 2017, S.H. He, He 5323 (BJFC 024841); Lushui County, Gaoligongshan Nature Reserve, on dead angiosperm branch, 28 November 2015, S.H. He, He 3293 (BJFC 021688); on dead *Ficus auriculata* tree, 30 November 2015, S.H. He, He 3410 (BJFC 021806); Mouding County, Huafo Mountains, on dead *Populus* branch, 25 November 2015, S.H. He, He 3139 (BJFC 021534); on angiosperm stump, 29 October 2017, S.H. He, He 5349 (BJFC 024867).

Substrate—usually on stumps of angiospermous trees.

Distribution—widely distributed in southern and northern China.

Notes—*Stratocorticium sinensis* is characterized by its strictly resupinate basidiomes with smooth to slight tuberculate hymenophores, a dark gray to black, stratified trama, a thickening subhymenium, micro-binding hyphae, brown skeletal-like hyphae, branched hyphidia, clavate to cylindrical cystidia with homogeneous contents, and small, capitate cystidia. Morphologically, *St. sinensis* and *Ce. gloeocystidiatum* share resupinate basidiomes, clamped generative and micro-binding hyphae, and small, cylindric basidiospores. The latter species differs in having smaller basidia and cylindric cystidia tapering to apex with golden yellow, homogeneous contents, and subfusiform hymenial cystidia with acute apices.

**A key to corticioid and stereoid genera in *Cyphellaceae***

1. Cystidia absent; basidiospores thick-walled  … … … … … … … … … … … … … … … … 2

1. Cystidia present; basidiospores thin-walled … … … … … … … … … … … … … … … … 3

2. Basidia plural; basidiospores distinctly ornamented; chlamydospores absent

 … … … … … … … … … … … … … … … … … … … … … … … … … …*Cunninghammyces*

2. Basidia terminal; basidiospores weakly ornamented; chlamydospores present

 … … … … … … … … … … … … … … … … … … … … … … … …… … … *Granulobasidium*

3. Basidiomes effused-reflexed to pileate  … … … … … … … … … … … … … … … … … …4

3. Basidiomes strictly resupinate … … … … … … … … … … … … … … … … … … … … … 5

4. Basidiomes gelatinous when fresh; with numerous gloeohyphae … … … … … *Gloeostereum*

4. Basidiomes coriaceous when fresh; without gloeohyphae … … … … … … …*Chondrostereum*

5. Acanthohyphidia and halocystidia present   … … … … … … … … … … …*Acanthocorticium*

5. Acanthohyphidia and halocystidia absent… … … … … … … … … … … … … … … … … 6

6. Cystidia of various shapes, often filled with dark yellow, resinous-like contents, context 

white … … … … … … … … … … … … … … … … … … … … … … … … … … … … *Cericium*

6. Cystidia clavate to cylindrical, contents homogenous, context dark gray to black

… … … … … … … … … … … … … … … … … … … … … … … … … … … … *Stratocorticium*

## 4. Discussion

The present paper intensively studied the corticioid and stereoid groups in *Cyphellaceae* for the first time. The phylogenetic relationship and taxonomic delimitation of different lineages are clarified with a new genus, *Stratocorticium*, introduced and *Cericium* placed. Our results indicate that *Cyphellaceae* has a high species diversity of corticioid fungi. Meanwhile, we provide the sequences of *Cunninghammyces umbonatus* to the public for the first time.

The *Agaricales* are dominated by mushrooms, but a number of corticioid, stereoid, or poroid wood-decay fungi are classified in the order because of molecular evidence. Moreover, these fungi are found in many lineages in various families, although only a few species are included in each lineage [12,42,43,44]. Our results support previous studies that demonstrate that *Cyphellaceae* contains corticioid and stereoid fungi [1,2,3,4]. Presently, seven corticioid and stereoid genera are classified in the *Cyphellaceae*, including *Cericium* and *Stratocorticium*, discussed above. The seven genera with corticioid or stereoid basidiomes display a wide range of morphologies relating to basidiome habit and texture, hymenophore types, hyphal systems, cystidium types, basidia shape and size, and basidiospore shape, size, and ornamentation. Without molecular data, it would be inconceivable to place the genera in the same family because there are so few morphological similarities among them.

The sampling of our phylogenetic analyses of *Cyphellaceae* was mainly based on previous results [1,4] that included many more genera than in other studies [25,45]. For most genera, only one or two species were included in the analyses above. Meanwhile, some of our samples lack the *ITS* sequences. The species diversity, taxonomy, and phylogeny of the *Cyphellaceae* are far from resolved, for the subtropic and tropic regions are understudied. Future phylogenetic studies in the *Cyphellaceae* would benefit from sequence data from multiple loci from a wide array of taxa from different regions of the world.

## Figures and Tables

**Table 1:** Taxa information and GenBank accession numbers of the sequences used in this study. New taxa are in bold with type specimens indicated with an asterisk (*).

Name	Voucher Number	Country	*ITS*	*nrLSU*	Reference
*Acanthocorticium brueggemannii*	JMB2621 ICN	Brazil	KT275194	KT275196	[3]
*Actiniceps laevis*	DJM1350	Singapore	—	DQ284917	[1]
*Atheniella amabilissima*	AFTOL-ID 1686	USA	DQ490644	DQ457691	[25]
*Atheniella aurantiidisca*	F15202 UBC	Canada	DQ384585	DQ384585	[1]
*Atheniella aurantiidisca*	AFTOL-ID 1685	USA	DQ490646	DQ470811	[25]
*Baeospora myosura*	AFTOL-ID 1799	USA	DQ484063	DQ457648	[25]
*Baeospora myriadophylla*	14-10-98-3	Not indicated	DQ868346	AF042634	[1]
*Calyptella capula*	AMB 18736	Italy	OL839159	—	[1]
*Calyptella capula*	PB315	Norway	AY571031	AY570995	[26]
*Campanophyllum proboscideum*	TENN56427	Mexico	AY230867	AY230867	[27]
*Campanophyllum proboscideum*	TENN56402	Mexico	AY230866	AY230866	[28]
*Cericium gloeocystidiatum*	He4332 *	**China**	**—**	**MW557949**	**This study**
*Cericium gloeocystidiatum*	**He4728**	**China**	**—**	**MW557950**	**This study**
*Cericium gloeocystidiatum*	**He20140723-8**	**China**	**—**	**MW557952**	**This study**
*Cericium gloeocystidiatum*	**He20140719-19**	**China**	**—**	**MW557951**	**This study**
*Chaetotyphula hyalina*	DJM1348	Singapore	—	DQ284912	[1]
*Cheimonophyllum candidissimum*	AFTOL-ID 1765	USA	—	DQ457654	[25]
*Cheimonophyllum candidissimum*	DAOM187959	USA	—	AF261357	[28]
*Chondrostereum purpureum*	He5634	China	MW557935	MW557942	This study
*Chondrostereum purpureum*	He6088	China	MW557936	MW557943	This study
*Chondrostereum purpureum*	LWZ 20201017-51	China	ON897843	ON885308	[29]
*Chondrostereum purpureum*	HHB-13334-sp.	USA	—	AF518607	[30]
*Cunninghammyces umbonatus*	He5311	China	MW557940	MW557954	This study
*Cunninghammyces umbonatus*	He5313	China	MW557941	—	This study
*Cunninghammyces umbonatus*	He5316	China	—	MW557955	This study
*Cyphella digitalis*	AFTOL-ID 663	USA	—	AY635771	[25]
*Cyphella digitalis*	Thorn-617	USA	—	AY293175	[1]
*Flammulina velutipes*	AFTOL-ID 558	Not indicated	AY854073	AY639883	[1]
*Gloeostereum cimri*	CBS 145006	USA	MT023735	MN266884	[31]
*Gloeostereum incarnatum*	KUC20131022-28	South Korea	KJ668540	KJ668393	[32]
*Gloeostereum incarnatum*	G1905	Russia	—	MK278092	[33]
*Gloeostereum incarnatum*	3332	Sweden	AF141637	AF141637	[34]
*Gloeostereum incarnatum*	BCC 41461	Thailand	KY614001	KY614002	[35]
*Granulobasidium vellereum*	CBS 214.54	France	MH857297	MH868829	[36]
*Granulobasidium vellereum*	CBS 124595	Netherlands	MH863395	MH874913	[36]
*Granulobasidium vellereum*	KUC20131022-21	South Korea	KJ668538	KJ668391	[37]
*Hemimycena gracilis*	AFTOL-ID 1732	USA	DQ490623	DQ457671	[25]
*Henningsomyces candidus*	PB338	France	AY571044	AY571008	[26]
*Hydropus paradoxus*	AMB 18772	Italy	OL839172	OL839185	[1]
*Lignomphalia lignicola*	LE262727	Russia	HM191731	—	[38]
*Mycenella bryophila*	15730/NL-3570	Italy	JF908500	MK278407	[33]
*Mycenella salicina*	843/KR 7435	France	JF908497	DQ071720	[1]
*Mycopan scabripes*	BRNM 793297	Czech Republic	OL839169	OL839183	[1]
*Mycopan scabripes*	TENN-F-076400	Canada	OL839163	OL839178	[1]
*Mycopan* sp.	TENN-F-076402	Canada	OL839171	OL839184	[1]
*Phloeomana clavata*	PRM 935560	Czech Republic	LT671447	LT671447	[1]
*Phloeomana speirea*	PRM 860810	Czech Republic	LT671446	LT671446	[1]
*Pleurella microspora*	AMB 18917	Italy	OL839173	OL839186	[1]
*Pleurella ardesiaca*	ICMP 20518	New Zealand	MH380192	MH380192	[1]
*Pleurotopsis longinqua*	RV95/473	Not indicated	—	AF042604	[28]
*Rectipilus idahoensis*	PB313/RA	France	AY571057	AY571020	[26]
*Scytinotus ringens*	Marson 30-Sep-2014	Luxembourg	KY007610	KY007610	[1]
*Setigeroclavula aff. Adscendens*	JAC9241	New Zealand	OL998338	—	[1]
*Stratocorticium sinensis*	He3289*	**China**	**MW557938**	**MW557953**	**This study**
*Stratocorticium sinensis*	**He4317**	**China**	**MW557939**	**MW557945**	**This study**
*Stratocorticium sinensis*	**He5323**	**China**	**MW557937**	**MW557944**	**This study**
*Stratocorticium sinensis*	**He2264**	**China**	**—**	**MW557946**	**This study**
*Stratocorticium sinensis*	**He4780**	**China**	**—**	**MW557947**	**This study**
*Stratocorticium sinensis*	**He2432**	**China**	**—**	**MW557948**	**This study**
*Stratocorticium sinensis*	**G1875**	**Russia**	**—**	**MK277899**	[39]

## Data Availability

The data presented in this study are openly available in GenBank (https://www.ncbi.nlm.nih.gov/, accessed on 31 January 2021) and Mycobank (https://www.mycobank.org/, accessed on 10 August 2024).
